# Dioxane dibromide mediated bromination of substituted coumarins under solvent-free conditions

**DOI:** 10.3762/bjoc.8.35

**Published:** 2012-02-29

**Authors:** Subrata Kumar Chaudhuri, Sanchita Roy, Sanjay Bhar

**Affiliations:** 1 Khorop High School, Howrah, West Bengal, India; 2 Department of Chemistry, Basanti Devi College, Kolkata – 700 029, India; 3 Department of Chemistry, Organic Chemistry Section, Jadavpur University, Kolkata – 700 032, India, Fax: 91-033-24141584

**Keywords:** coumarins, solvent-free reaction, substituents, vinylic bromination

## Abstract

An efficient solvent-free protocol for regioselective bromination of substituted coumarins has been developed by using dioxane dibromide as the solid brominating agent. The efficacy of the solvent-free protocol has been established. The effects of the electronic nature and location of the substituents on the outcome of the reaction have been rationalized with a proposed mechanism.

## Introduction

Brominated coumarins have immense synthetic, biological and industrial importance due to their occurance in nature [1] and applications in pharmaceuticals. They are used for the synthesis of formyl [2] and aryl derivatives [3] and serve as important synthetic precursors of furocoumarins and dihydrofurocoumarins, which are widely used as photosensitizers and chemotherapeutic agents to combat skin diseases [4]. Halocoumarins also exhibit insecticidal and fungicidal properties [5]. A few methods have been documented for regioselective bromination of coumarins, which include CuBr_2_/Al_2_O_3_ in bromobenzene under reflux [5], Br_2_ in glacial AcOH [6], Br_2_/Al_2_O_3_ under microwave irradiation [7], NBS in CHCl_3_ [8], Et_4_N^+^Br^−^ in the presence of hypervalent iodine reagents [9], and NBS in tetrabutylammonium bromide under molten salt conditions [10]. There is a recent report of the preparation of 3-bromocoumarins from acyclic precursors through bromination of a Wittig reagent with NBS followed by tandem Wittig reaction and cyclization [11]. Many of them involve high temperature, long reaction time, toxic, exotic and costly reagents, sometimes in excess to stoichiometric requirement, and organic solvents during and after the reactions. Thus, there is always an urgency to put ardent efforts towards the development of new methods for regioselective bromination of differently substituted coumarins under mild conditions with easily accessible reagents. Dioxane dibromide (DD) has been successfully used for selective α-bromination of substituted acetophenones by using dioxane as a solvent at room temperature [12], and for the selective synthesis of α-bromo and α,α-dibromoalkanones supported on silica gel under solvent-free conditions and microwave irradiation [13]. As a part of our endeavor to develop novel solvent-free protocols for important organic transformations, we have reported the efficient synthesis of vicinal *anti*-dibromides through highly diastereoselective electrophilic addition of bromine across various electron-deficient and electron-rich double bonds [14] using DD under solvent-free conditions. Two compounds of the present investigation (**2a** and **2g** in Table 1, see below) were synthesized during the aforesaid study [14]. We have also accomplished DD-mediated solvent-free regioselective electrophilic ring bromination of aromatic compounds [15]. Encouraged by the aforesaid literature precedence we envisaged the development of a solvent-free protocol for regioselective bromination of substituted coumarins using DD as an easily accessible, economically viable reagent, and we report herein the results of our investigation.

## Results and Discussion

**Scheme 1:**
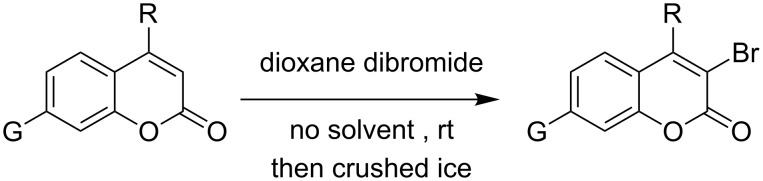
Dioxane dibromide-mediated solvent-free bromination of substituted coumarins.

When dioxane dibromide (DD, an orange solid compound, mp 61–62 °C, molecular formula C_4_H_8_O_2_Br_2_ containing 63.6% of bromine as determined iodometrically) was thoroughly mixed with the substrate in the absence of any solvent, kept at room temperature for the stipulated time and triturated with crushed ice, then, depending on the stoichiometric proportion of DD, the respective product was obtained as a filterable solid. This is delineated in Scheme 1 and the detailed results are summarized in Table 1.

**Table 1:** Solvent-free reactions of substituted coumarins with dioxane dibromide.

Entry^a^	Substrate	Molar equiv DD	Product	Time (h)	Yield (%)^b^

1	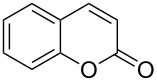 **1a**	1.2	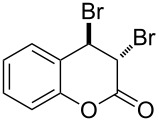 **2a**	0.5	76 [14,16]
2	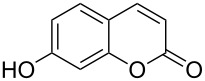 **1b**	1.0	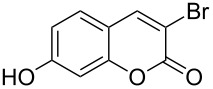 **2b**	2.0	79^c^ [5,10]
3	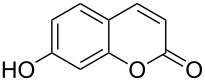 **1b**	2.2	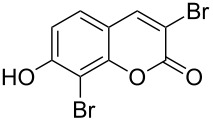 **2bb**	4.0	72^c^ [11]
4	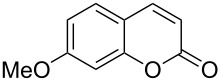 **1c**	1.2	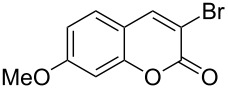 **2c**	3.0	79 [17]
5	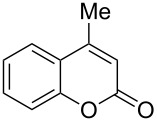 **1d**	1.2	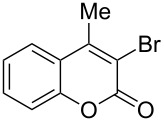 **2d**	2.0	83 [6]
6	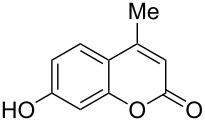 **1e**	1.2	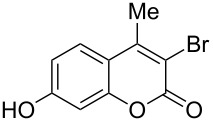 **2e**	3.0	84^c^ [5,8]
7	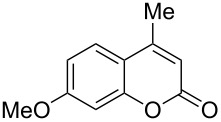 **1f**	1.2	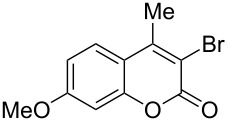 **2f**	2.0	85 [8,10]
8	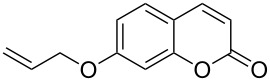 **1g**	1.2	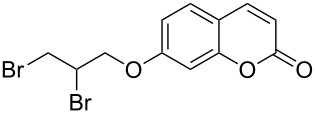 **2g**	0.5	83 [14]
9	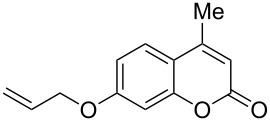 **1h**	1.2	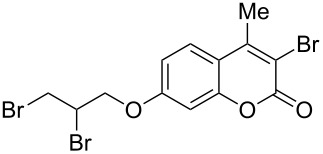 **2h**	4.0	56
10	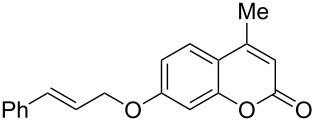 **1i**	2.2	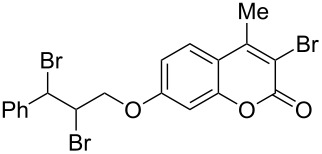 **2i**	4.0	86
11	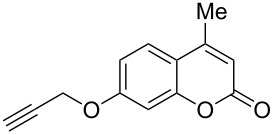 **1j**	2.2	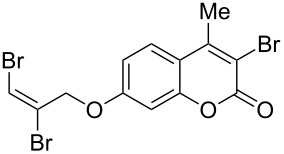 **2j**	2.0	73
12	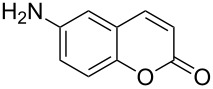 **1k**	0.7	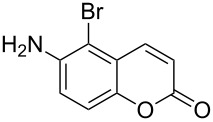 **2k**	1.0	62 [10]
13	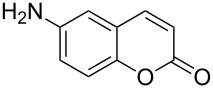 **1k**	2.2	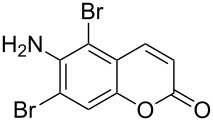 **2kk**	1.0	70 [18]
14	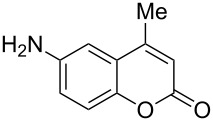 **1l**	2.2	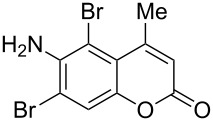 **2l**	2.0	77

^a^Reactions were carried out with 5 mmol of substrate by using the duly weighed amount of DD as indicated by the molar equiv; ^b^yield refers to that of the pure product characterized spectroscopically; ^c^products were characterized through the acetyl derivative.

When an unsubstituted coumarin (**1a**) was subjected to bromination with DD under solvent-free conditions, vicinally *anti*-dibromide **2a** was obtained exclusively through electrophilic addition of bromine across the electron-deficient double bond of the conjugated δ-lactone moiety (Table 1, entry 1). This was evident from the dihedral angle between H–C3–C4 and C3–C4–H in **2a**, which was calculated (from the Karplus equation) as 57.5° from the coupling constant (2.6 Hz) between C3–H and C4–H at δ 4.96 and δ 5.34. However, for the aromatic ring substituted with the electron-donating group at C7, the heterocyclic ring in **1b** and **1c** underwent selective monobromination with an equimolar amount of DD through vinylic substitution of C3–H. In the ^1^H NMR spectra of **2b** and **2c,** the signal around δ 6.00 was absent and one singlet around δ 8.00 appeared due to olefinic hydrogen at C4 having no partner to couple with. Thus the incorporation of bromine at C3 (Table 1, entries 2 and 4) was established. When **1b** reacted with more than two equivalents of DD, the second bromine atom was incorporated in the carbocyclic ring at C8 through aromatic electrophilic substitution (Table 1, entry 3) to produce **2bb**. The location of the second bromine atom in **2bb** was also established conclusively from its ^1^H NMR spectrum in which only two doublets with coupling constants of 8.4 Hz appeared due to aromatic hydrogens (apart from the singlet at δ 8.07 due to olefinic-H at C4) at δ 7.13 and 7.43, representing two *ortho* protons, which were bound to be due to aromatic hydrogens at C6 and C5, respectively. Obviously C8 carried the second bromine atom in **2bb**. In **1d**, with an alkyl substituent at C4, vinylic bromination occurred at C3 (Table 1, entry 5). This was supported from ^1^H NMR data in which the singlet at δ 6.29 due to the hydrogen at C3 of **1d** was absent in **2d** indicating vinylic substitution. In the ^13^C NMR spectrum a signal appeared at δ 151.0 due to the quaternary C carrying the bromine atom. When C4 and C7 were both substituted with an alkyl group and electron-donating group, respectively, as in **1e** and **1f**, vinylic bromination occurred regioselectively to furnish **2e** and **2f** in high yield without further bromination at C6 and C8, when a stoichiometric amount of DD was used (Table 1, entries 6 and 7).

Interestingly, 7-(allyloxy)coumarin (**1g**) underwent chemoselective addition of bromine across the isolated double bond to form **2g** leaving behind the conjugated endocyclic double bond unaffected, when a critically weighed amount of DD as per stoichiometric requirement was used (Table 1, entry 8). This was supported by the ^1^H NMR data of **2g** in which the doublet for olefinic H at C3 appeared at δ 6.27 with *J* = 9.4 Hz and olefinic hydrogen at C4 appeared at δ 7.65 with *J* = 9.5 Hz, corresponding to the mutually *cis*-orientation of the two hydrogens across the C3–C4 olefinic linkage of the coumarin system. Three peaks in the ratio 1:2:1 as the molecular ion peaks in the mass spectrum also conclusively proved the presence of two bromine atoms in the product, which were due to electrophilic addition of bromine across the olefinic linkage without any vinylic substitution. 4-Methyl-7-(allyloxy)coumarin (**1h**) (Table 1, entry 9) underwent addition of bromine across the isolated double bond as well as vinyl substitution at C3, in contrast to our previous result (Table 1, entry 8). This indicated a strong influence of the alkyl substituent at C4 on the course of the reaction. The results for cinnamyloxy and propargyloxy-substituted 4-methylcoumarins (**1i** and **1j)** were in accordance with the previous result (Table 1, entries 10 and 11).

It is important to note that the presence of an electron-donating group (–NH_2_) at C6, without an alkyl substituent at C4, lead to bromination of the carbocyclic ring bearing the amino group through aromatic electrophilic substitution instead of vinylic bromination, leaving the conjugated double bond intact (Table 1, entry 12). This was established by the presence of two doublets (*J* = 9.6 Hz) at δ 6.46 and δ 8.03 in **2k** due to olefinic H’s at C3 and C4, respectively, indicating the *cis*-stereochemistry of the olefinic linkage of the α,β-unsaturated-δ-lactone moiety. Two other doublets (*J* = 9.0 Hz) at δ 6.95 and δ 7.15 appeared due to *ortho*-coupled aromatic hydrogens at C7 and C8, indicating incorporation of bromine at C5. In the presence of excess DD, **1k** and **1l** furnished 5,7-dibromo derivatives **2kk** and **2l**, respectively, through two successive electrophilic aromatic substitutions at the more activated carbocyclic aromatic ring without vinylic bromination, regardless of the presence or absence of the alkyl substituent at C4 (Table 1, entries 13 and 14). The appearance of only one singlet around δ 7.5 (due to aromatic-H at C8) in **2kk** and **2l** indicated the incorporation of two bromine atoms at C5 and C7, which were the more nucleophilic centers as both of them were *ortho* with respect to the electron-donating amino group.

**Scheme 2:**
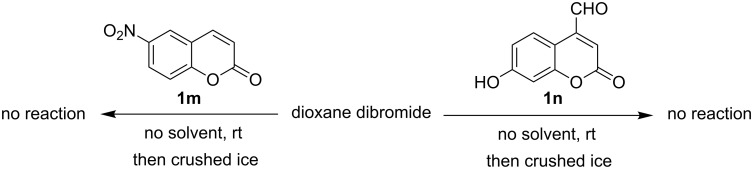
Failure of DD to react with coumarins substituted with an electron-withdrawing group.

From the aforesaid study, it is clear that the outcome of this dioxane dibromide mediated solvent-free bromination reaction depends critically on the electronic nature and location of the substituents. This was further corroborated from the fact that coumarins bearing electron-withdrawing substituents (**1m** and **1n**) remained unaffected under the applied reaction conditions (Scheme 2).

**Scheme 3:**
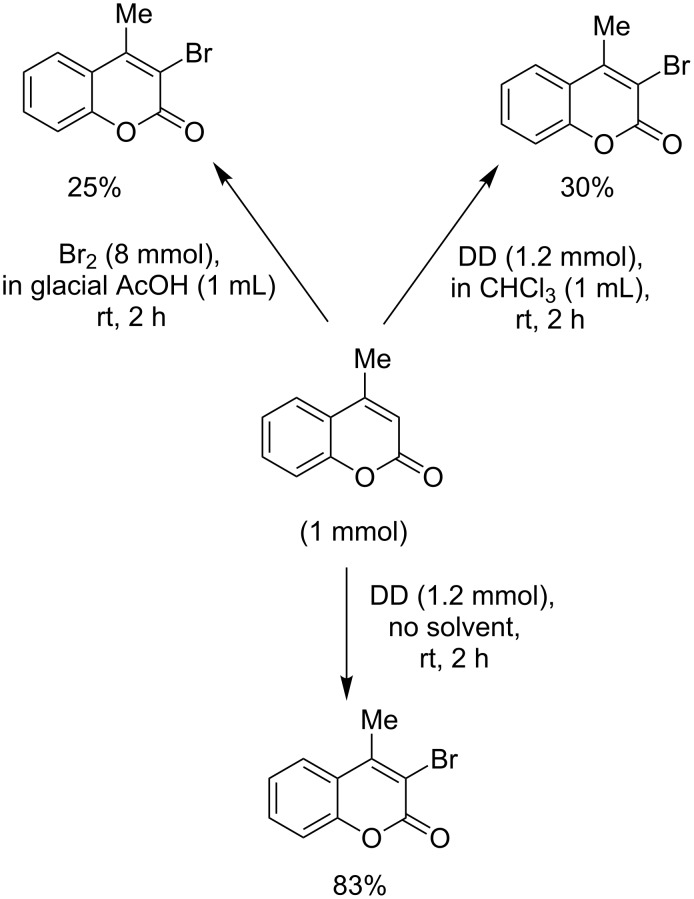
Efficacy of the solvent-free bromination protocol with DD.

The above-mentioned protocol avoids the use of molecular bromine [19], in halogenated or other toxic organic solvents [5,8,19], and any kind of inorganic support [5]. Thus, synthetically important 3-bromo-umbelliferone (**2b**) and its derivatives, used as precursors for the synthesis of sulfones with remarkable antituberculotic activity [20] or other biologically important molecules [20–21] can be accessed easily by this simple protocol. For example, 3,8-dibromo-7-hydroxycoumarin (**2bb**) has come out as the most promising inhibitor of casein kinase 2 [22]. In this protocol, excess use of toxic bromine can be avoided by using the requisite amount of solid brominating agent through accurate weighing. A stoichiometric amount of HBr is liberated, but this is less toxic than Br_2_ vapour. Therefore, this solvent-free protocol for the bromination of coumarins helps to minimize the involvement and dispersal of harmful chemicals in the environment. Moreover, DD under solvent-free conditions is a superior reagent for the bromination of coumarins in comparison to bromine in an organic solvent, as shown in Scheme 3. DD reacts with coumarins faster and more efficiently in the absence of solvent. Considerable amounts of unidentified byproducts were produced when bromine and DD were used in organic solvents. Therefore, the yield and purity of the product were depleted. The efficacy of the dioxane dibromide mediated solvent-free protocol has thus been clearly established in terms of reaction rate, yield and purity of the products as well as the ease of accessibility of the reagent along with procedural simplicity.

**Figure 1:**
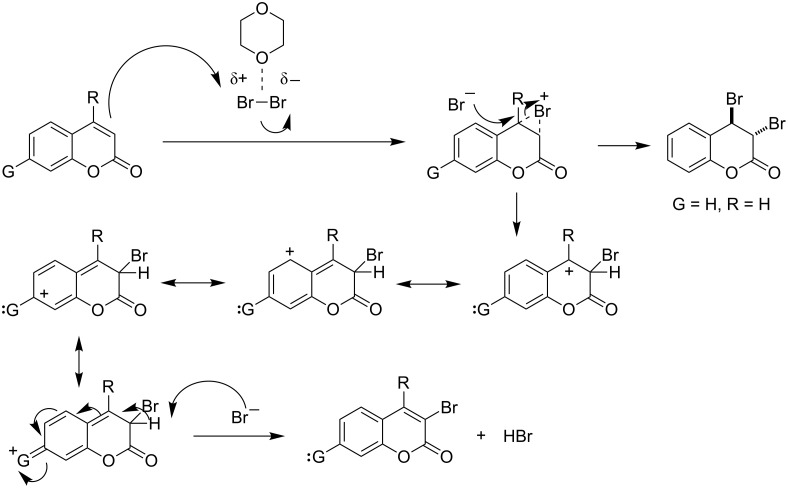
Mechanism for bromination of the heterocyclic ring.

From the aforesaid study it is clear that differentially substituted coumarins undergo transformations involving vinylic bromination and/or ring bromination, which are critically dictated by the electronic nature and location of the substituent(s). A plausible mechanism has been proposed to rationalize the electronic and positional effects of the substituents on the course of the reaction (Figure 1). The ability of the substituents to stabilize the electron-deficient centers seems to play the most crucial role in determining the reactivity and orientation of the said reaction.

The orange colour of DD is believed to originate from a charge-transfer transition between dioxane and bromine [23]. It has been reported [24] that the Br–Br bond length in DD is 0.231 nm, whereas in the Br_2_ molecule, the Br–Br bond length is 0.228 nm. Thus, the Br–Br bond in DD is a little elongated and the bromine molecule is slightly polarized. It has also been suggested [24] that when bromine interacts with dioxane forming a dioxane dibromide complex, the electron density flows from the lone pair of nonbonding occupied orbitals of bromine to the unoccupied orbitals on the dioxane unit. Therefore, the attacking electrophile derived from DD is likely to be a slightly polarized neutral bromine molecule with enhanced electrophilicity. As proposed in Figure 1, the reaction may involve the initial formation of a cyclic bromonium ion and its subsequent conversion to the carbocationic intermediate, depending on its stabilization by alkyl substituents at C4 through an electron-donating inductive effect and hypercojugation, and/or by the substituents at C7 rendering an electron-donating mesomeric effect. The formation of the initial bromonium ion intermediate was ascertained by the highly antiselective addition of bromine across the electron-deficient olefinic bond of the unsubstituted coumarin (Table 1, entry 1). In other cases, the bromonium ion underwent ring opening such that the incipient positive charge at C4 was stabilized by an alkyl group present at C4, through an electron-donating inductive effect and hyperconjugation as well as the mesomeric effect of the electron-donating substituent at C7. After concomitant loss of highly acidic hydrogen from C3, the carbocationic intermediate was re-aromatized leading to vinylic substitution (Table 1, entries 2–7). It is interesting to note that the electron-rich isolated double bond underwent preferential addition of bromine, rather than vinylic substitution at C3, when there was no alkyl substituent at C4 (Table 1, entry 8). But when the C4 had an alkyl substituent, the isolated double bond underwent bromine addition along with vinylic substitution at C3, despite the use of 1 molar equivalent of DD (Table 1, entry 9). Thus, the importance of the alkyl substituent at C4 towards vinylic substitution was established. Hence, the electrophilic addition and vinylic bromination could occur at the same time by using 2 molar equivalents of DD (Table 1, entry 10). Acetylenic bonds, being more nucleophilic than olefinic bonds, underwent addition of bromine to vicinal dibromo-olefins along with vinylic substitution (Table 1, entry 11). Further bromination did not occur due to a less pronounced nucleophilicity of the brominated olefinic linkage in **2j**.

**Figure 2:**
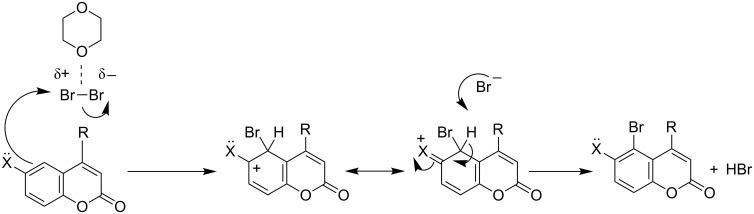
Mechanism for bromination of the carbocyclic ring.

The presence of an electron-donating substituent at C6 increased the electron density to the carbocyclic ring at the adjacent carbons. Moreover, the electron-donating substituent at C6 could not stabilize the Wheland intermediate (obtained by the electrophilic attack at C3) as efficiently as that at C7 through mesomeric delocalization. Rather, it was more capable of stabilizing the positive charge in the Wheland intermediate obtained after the electrophilic attack at C5, as shown in Figure 2. Thus, bromination at C5 and C7 occurred through aromatic electrophilic substitution of the carbocyclic ring rather than vinylic bromination (Table 1, entries 12–14) at the heterocyclic ring.

When C4 was substituted with an electron-withdrawing group, e.g., –CHO, the development of positive charge at that position was highly disfavored. Therefore, the reaction did not proceed and the starting material was recovered unchanged (Scheme 2). The electron-withdrawing substituents (e.g., –NO_2_) on the carbocyclic ring lowered its electron-density. Hence, the incipient positive charge at C4 could not be stabilized by mesomeric delocalization. Therefore, the reaction failed (Scheme 2) with electron-withdrawing substituents.

## Conclusion

The present dioxane dibromide mediated solvent-free protocol provides a very simple, versatile and efficient bromination of differentially substituted coumarins. The most significant features of this methodology are (a) good accessibility of the reagent and its stability (stable for at least three to four months in a refrigerator); (b) a stoichiometric amount of reagent can be used by direct weighing, avoiding excess; (c) no evolution of hazardous bromine vapor during the reaction; (d) the total elimination of the use of toxic organic solvents; (e) a simple experimental procedure; (g) an easy work-up; (h) excellent selectivity in case of activated coumarins and (i) good control over the outcome of the reaction by varying the amount of reagent. The aforesaid protocol thus provides an improved procedure for the synthesis of useful bromocoumarins having important pharmaceutical, agricultural and other physicochemical properties.

## Supporting Information

File 1 General experimental procedure for the preparation of DD and solvent-free bromination of substituted coumarins using DD along with the spectral data of the products.
