# Supplementary material for: Dioxane dibromide mediated bromination of substituted coumarins under solvent-free conditions
Source: Beilstein J Org Chem. 2012 Feb 29;8:323–9. doi: 10.3762/bjoc.8.35 (PMC3302096; doi:10.3762/bjoc.8.35)
Supplement: File 1 — General experimental procedure for the preparation of DD and solvent-free bromination of substituted coumarins using DD along with the spectral data of the products. [file Beilstein_J_Org_Chem-08-323-s001.pdf]

Supporting Information

for

Dioxane dibromide mediated bromination of substituted coumarins under solvent-free conditions

Subrata Kumar Chaudhuri[1], Sanchita Roy[2] and Sanjay Bhar[*3]

Address: [1]Khorop High School, Howrah, West Bengal, India, [2]Department of Chemistry, Basanti Devi College, Kolkata – 700 029, India and [3]Department of Chemistry, Organic Chemistry Section, Jadavpur University, Kolkata – 700 032, India, Fax: 91-033-24141584

E-mail: Sanjay Bhar - sanjaybharin@yahoo.com

*Corresponding author

General experimental procedure for the preparation of DD and solvent-free bromination of substituted coumarins using DD along with the spectral data of the products.

Preparation of dioxane dibromide [1]**:**

Dioxane dibromide was prepared following the reported procedure [1] with a slight modification. Bromine (3 ml, 9.3 g, 58.1 mmol) was added dropwise to ice-cold dioxane (8 ml, 8.1 g, 92 mmol) under stirring upon which an orange-coloured solid appeared. After complete addition, the reaction mixture was kept at rt for a further 2 h. The orange product was filtered, washed with dioxane, dried in a desiccator under reduced pressure (yield 9.3 g, 65%) and stored in a refrigerator at below 0 °C for several months.

General procedure for dioxane dibromide-mediated solvent-free bromination of substituted coumarins (adapted from [2])**:**

The general procedure followed the earlier literature [2]. Dioxane dibromide (as required by the stoichiometry mentioned in the main manuscript, Table 1) was added in portions to the neat substrate (5 mmol, cooled to 0–5 °C with ice-water) in an open vessel fitted with a $CaCl_2$ drying tube and thoroughly mixed with a spoon-headed glass rod. The mixture was allowed to reach ambient temperature and left standing for the indicated time (Table 1) under anhydrous conditions. The entire operation was carried out inside a fume cupboard to drive out the liberated hydrogen bromide fumes. Crushed ice was then added to the reaction mixture, stirred well and allowed to settle. The solid product was filtered, washed successively with saturated aqueous sodium bicarbonate solution and water, and dried to get the product in an almost pure form. The products were purified by column chromatography on silica gel or alumina followed by crystallization.

Spectral and analytical data of substituted bromocoumarins:

***trans*-3,4-Dibromo-3,4-dihydrocoumarin (2a) (Table 1, entry 1)**

Crystallized from diethyl ether/hexane; mp 94–97 °C; yield 76%; IR (KBr): 1735 cm^{-1} (s), ^{1}H NMR (300 MHz, $CDCl_3$) δ 4.96 (1H, d, J = 2.6 Hz), 5.34 (1H, d, J = 2.6 Hz), 7.17 (1H, d, J = 8.1 Hz), 7.25 (1H, dt, J_1 = 7.9 Hz, J_2 = 1.9 Hz), 7.38 (1H, dd, J_1 = 8.0 Hz, J_2 = 1.5 Hz), 7.45 (1H, dt, J_1 = 7.9 Hz, J_2 = 1.8 Hz); Anal. calcd for $C_9H_6O_2Br_2$: C, 35.31; H, 1.97; found: C, 35.25; H, 1.73.

3-Bromo-7-hydroxycoumarin (2b) (Table 1, entry 2):

This compound was obtained as a semisolid mass (yield 79%), which solidified on cooling and standing. Compound **2b** was characterized as its acetate derivative (treating with acetic anhydride/sodium acetate) and crystallized from diethyl ether/hexane; mp 133–134 °C; ^{1}H NMR (300 MHz, $CDCl_3$) δ 2.34 (3H, s), 7.09 (1H, dd, J_1 = 8.5 Hz, J_2 = 1.9 Hz), 7.14 (1H, d, J = 1.9 Hz), 7.46 (1H, d, J = 8.4 Hz), 8.08 (1H, s).

3,8-Dibromo-7-hydroxycoumarin (2bb) (Table 1, entry 3):

This compound was obtained as a gummy solid (yield 72%) and characterized (^{1}H NMR) as the corresponding acetate derivative (crystallized from dichloromethane/hexane); mp 147–149 °C; ^{1}H NMR (300 MHz, $CDCl_3$) δ 2.40 (3H, s), 7.13 (1H, d, J = 8.3 Hz), 7.43 (1H, d, J = 8.4 Hz), 8.07 (1H, s); Anal. calcd for $C_{11}H_6O_4Br_2$: C, 36.50; H, 1.67; found: C, 36.36; H, 1.74.

3-Bromo-7-methoxycoumarin (2c) (Table 1, entry 4):

Purified by column chromatography on silica gel with 5% ethyl acetate-hexane as eluent followed by recrystallization from acetone/hexane; yield 79%; mp 157–158 °C; ^{1}H NMR (300 MHz, $CDCl_3$) δ 3.88 (3H, s), 6.81 (1H, d, J = 2.1 Hz), 6.87 (1H, dd, J_1 = 8.7 Hz, J_2 = 2.1 Hz), 7.35 (1H, d, J = 8.7 Hz), 8.02 (1H, s); ^{13}C NMR (75 MHz, $CDCl_3$) δ 55.9, 100.8, 107.8, 113.3, 128.1, 143.2, 144.5, 155.1, 157.5, 163.0.

3-Bromo-4-methylcoumarin (2d) (Table 1, entry 5):

Purified by column chromatography with 10% ethyl acetate/hexane as eluent; yield 83%; mp 112 °C; FTIR (KBr): 1723.74, 1597.76, 1446.94, 973.50, 768.26 cm^{-1}; ^{1}H NMR (300 MHz, $CDCl_3$) δ 2.62 (3H, s), 7.33 (2H, t, J = 8.1 Hz), 7.56 (1H, t, J = 8.1 Hz), 7.65 (1H, d, J = 8.1 Hz); ^{13}C NMR (75 MHz, $CDCl_3$) δ 19.5, 113.1, 116.9, 119.6, 124.9, 131.9, 151.0, 151.7, 156.8.

3-Bromo-7-hydroxy-4-methylcoumarin (2e) (Table 1, entry 6)

Purified by column chromatography on silica gel with 10% ethyl acetate/hexane as eluent; yield 84%, mp 210–212 °C. Compound **2e** was characterized as its acetate derivative; mp 148–150 °C; ^{1}H NMR (300 MHz, $CDCl_3$) δ 2.35 (3H, s), 2.63 (3H, s), 7.11 (1H, d, J = 2.1 Hz), 7.15 (1H, distorted t, J = 2.6 Hz), 7.67 (1H, d, J = 8.6 Hz).

3-Bromo-7-methoxy-4-methylcoumarin (2f) (Table 1, entry 7)

Purified by filtration chromatography on neutral alumina with 5% ethyl acetate/hexane as eluent followed by recrystallization from ethyl alcohol; yield 85%; mp 127–128 °C; ^{1}H NMR (300 MHz, $CDCl_3$) δ 2.58 (3H, s), 3.87 (3H, s), 6.80 (1H, d, J = 1.9 Hz), 6.88 (1H, dd, J_1 = 8.9 Hz, J_2 = 2.0 Hz), 7.54 (1H, d, J = 9.0 Hz).

7-(2,3-Dibromopropoxy)coumarin (2g) (Table 1, entry 8):

Purified by column chromatography on neutral alumina with 7% ethyl acetate/hexane as eluent followed by recrystallization from ether/petroleum ether; yield 83%; mp 103–105 °C; IR(KBr): 1720cm^{-1} (s); ^{1}H NMR (300 MHz, $CDCl_3$) δ 3.89–3.92 (2H, m), 4.42–4.48 (3H, m), 6.27 (1H, d, J = 9.4 Hz), 6.84 (1H, d, J = 2.3 Hz), 6.89 (1H, dd, J_1 = 8.6 Hz, J_2 = 2.3 Hz), 7.41 (1H, d, J = 8.6 Hz), 7.65 (1H, d, J = 9.5 Hz); ^{13}C NMR (75 MHz, $CDCl_3$) δ 32.1, 46.7, 69.3, 101.9, 112.7, 113.3, 113.7, 128.9, 143.2, 155.6, 160.8, 160.9; Anal. calcd for $C_{12}H_{10}O_3Br_2$: C, 39.78; H, 2.78; found: C, 39.66, H, 2.73; HRMS (*m*/*z*): $[C_{12}H_{10}O_3Br_2 + Na]^+$ calcd for: 382.8896, 384.8876, 386.8856; found: 382.8894, 384.8875, 386.8856 in a ratio 1:2:1.

3-Bromo-7-(2,3-dibromopropoxy)-4-methylcoumarin (2h) (Table 1, entry 9):

Purified by column chromatography on neutral alumina with 6% ethyl acetate/hexane as eluent followed by crystallization from ethyl alcohol; yield 56%; mp 127–129 °C, ^{1}H NMR (300 MHz, $CDCl_3$) δ 2.60 (3H, s), 3.91 (2H, distorted d, J = 6.3 Hz), 4.43–4.46 (3H, m), 6.86 (1H, d, J = 2.4 Hz), 6.95 (1H, dd, J_1 = 9.0 Hz, J_2 = 2.3 Hz), 7.58 (1H, d, J = 9.0 Hz); ^{13}C NMR (75 MHz, $CDCl_3$) δ 19.6, 32.2, 46.7, 69.5, 101.8, 110.3, 113.2, 114.1, 126.3, 151.0, 153.4, 157.2, 160.9; Anal. calcd for $C_{13}H_{11}O_3Br_3$: C, 34.32; H, 2.43; found: C, 34.45; H, 2.29.

3-Bromo-7-(2,3-dibromo-3-phenylpropoxy)-4-methylcoumarin (2i) (Table 1, entry 10):

Purified by column chromatography on neutral alumina with 10% ethyl acetate/hexane as eluent followed by recrystallization from acetone; yield 86%. ^{1}H NMR (300 MHz, $CDCl_3$) δ 2.61 (3H, s), 4.61 (1H, distorted dd, J_1 = 9.9 Hz, J_2 = 3.1 Hz), 4.76–4.85 (2H, m), 5.41 (1H, d, J = 10.0 Hz), 6.90 (1H, d, J = 2.2 Hz), 7.01 (1H, dd, J_1 = 8.8 Hz, J_2 = 2.1 Hz), 7.33–7.45 (5H, m), 7.61 (1H, d, J = 8.9 Hz); ^{13}C NMR (75 MHz, $CDCl_3$) δ 19.7, 52.4, 52.5, 71.4, 102.0, 110.4, 113.5, 114.3, 126.4, 128.0, 128.9, 129.3, 139.2, 151.0, 153.6, 157.3, 161.2; HRMS (*m*/*z*): $[C_{19}H_{15}O_3Br_3 + Na]^+$ calcd for 550.8470, 552.8451, 554.8431, 556.8411; found, 550.8469, 552.8449, 554.8430, 556.8414 in a ratio of 1:3:3:1.

3-Bromo-7-(2,3-dibromoallyloxy)-4-methylcoumarin (2j) (Table 1, entry 11):

Purified by column chromatography on silica gel with 10% ethyl acetate/hexane as eluent followed by recrystallization from dichloromethane/hexane; yield 73%; mp 120 °C; FTIR (KBr): 3087.32, 1722.83, 1611.10, 1295.15, 1151.64, 751.78 cm^{-1}; ^{1}H NMR (300 MHz, $CDCl_3$) δ 2.58 (3H, s), 4.94 (2H, s), 6.77 (1H, s), 6.85 (1H, d, J = 2.1 Hz), 6.94 (1H, dd, J_1= 9.0 Hz, J_2 = 2.1 Hz), 7.58 (1H, d, J = 9.0 Hz); ^{13}C NMR (75 MHz) δ 19.4, 68.4, 102.0, 107.4, 111.0, 113.6, 114.1, 119.9, 126.1, 150.8, 153.2, 157.0, 160.3.

6-Amino-5-bromocoumarin (2k) (Table 1, entry 12):

Purified by crystallization from dichloromethane/hexane; yield 62%; mp 168–169 °C; IR (KBr): 3456, 3331, 1708, 1623, 1559, 813 cm^{-1}; ^{1}H NMR (300 MHz, $CDCl_3$) δ 4.17 (2H, broad- s), 6.46 (1H, d, J = 9.6 Hz), 6.95 (1H, d, J = 9.0 Hz), 7.15 (1H, d, J = 9.0 Hz), 8.03 (1H, d, J = 9.6 Hz).

6-Amino-5,7-dibromocoumarin (2kk) (Table 1, entry 13):

Purified by crystallization from dichloromethane/hexane; yield 70%; mp 197–198 °C; ^{1}H NMR (300 MHz, $CDCl_3$) δ 4.64 (2H, s), 6.49 (1H, d, *J* = 9.9 Hz), 7.50 (1H, s), 7.98 (1H, d, *J* = 9.9 Hz); Anal. calcd for $C_9H_5O_2NBr_2$: C, 34.10; H, 1.59; N, 4.41; found: C, 34.24; H, 1.68; N, 4.31.

6-Amino-5,7-dibromo-4-methylcoumarin (2l) (Table 1, entry 14):

Purified by crystallization from dichloromethane/hexane; yield 77%; mp >250 °C; FTIR (KBr): 3427.93, 3322.68, 1747.43, 1732.47, 1598.61, 975.94, 884.55cm^{-1}; ^{1}H NMR (300 MHz, $CDCl_3$) δ 2.81 (3H, s), 4.61 (2H, s), 6.33 (1H, s), 7.52 (1H, s); HRMS (*m*/*z*): $[C_{10}H_7O_2NBr_2 + Na]^+$ calcd for 353.8743, 355.8723, 357.8703; found: 353.8793, 355.8722, 357.8706 in a ratio 1:2:1.

References:

1. Juneja, S. K.; Gupta, M.; Paul, S.; Gupta, R. *Bull. Korean Chem. Soc.* **2008**, *29*, 2337–2340.
2. Chaudhuri, S. K.; Roy, S.; Saha, M.; Bhar, S. *Synth.Commun.* **2007**, *37*, 579–583.
